# Long-term prognostic significance of HER-2/*neu *in untreated node-negative breast cancer depends on the method of testing

**DOI:** 10.1186/bcr991

**Published:** 2005-01-26

**Authors:** Marcus Schmidt, Barbara Lewark, Nikolai Kohlschmidt, Christiane Glawatz, Erik Steiner, Berno Tanner, Henryk Pilch, Wolfgang Weikel, Heinz Kölbl, Hans-Anton Lehr

**Affiliations:** 1 Department of Obstetrics and Gynecology, Johannes Gutenberg University, Medical School, Mainz, Germany; 2 Department of Pathology, Johannes Gutenberg University, Medical School, Mainz, Germany

**Keywords:** breast cancer, FISH, HER-2/neu, node-negative, prognosis

## Abstract

**Introduction:**

The prognostic significance of HER-2/*neu *in breast cancer is a matter of controversy. We have performed a study in 101 node-negative breast cancer patients with long-term follow-up not treated in the adjuvant setting, and analysed the prognostic significance of immunohistochemistry (IHC) and fluorescence *in situ *hybridisation (FISH), both separately and in combination, in comparison with traditional prognostic factors.

**Methods:**

Overexpression was classified semiquantitatively according to a score (0 to 3+) (HER-2_SCO). FISH was used to analyse HER2/*neu *amplification (HER-2_AMP). Patients classified 2+ by IHC were examined with FISH for amplification (HER-2_ALG). Patients with 3+ overexpression as well as amplification of HER-2/*neu *were positive for the combined variable HER2_COM. These variables were compared with tumour size, histological grade and hormone receptor status.

**Results:**

HER-2_SCO was 3+ in 20% of all tumours. HER-2_ALG was positive in 22% and amplification (HER-2_AMP) was found in 17% of all tumours. Eleven percent of the tumours showed simultaneous 3+ overexpression and amplification. Only histological grade (relative risk [RR] 3.22, 95% confidence interval [CI] 1.73–5.99, *P *= 0.0002) and HER-2_AMP (RR 2.47, 95% CI 1.12–5.48, *P *= 0.026) were significant for disease-free survival in multivariate analysis. For overall survival, both histological grade (RR 3.89, 95% CI 1.77–8.55, *P *= 0.0007) and HER-2_AMP (RR 3.08, 95% CI 1.24–7.66, *P *= 0.016) retained their independent significance.

**Conclusion:**

The prognostic significance of HER-2/*neu *in node-negative breast cancer depends on the method of testing: only the amplification of HER-2/*neu *is an independent prognostic factor for the long-term prognosis of untreated node-negative breast cancer.

## Introduction

Human epidermal growth factor receptor-2 is a proto-oncogene that encodes a cell-surface receptor designated HER-2/*neu *or c-erbB-2. Gene amplification and/or protein overexpression occurs in 14–30% of all breast cancers [1-3]. Initially, the adverse prognostic impact of HER-2/*neu *in breast cancer was the main focus of research. However, results from different study groups were not entirely consistent. Studies that supported the initially reported adverse prognosis in breast cancer [1,3] were later followed by reports that failed to show any association with prognosis [4,5]. Although no consensus exists concerning the prognostic value of HER-2/*neu*, an increasing quantity of data indicates a predictive value for the efficacy of certain adjuvant therapies. The response of HER-2/*neu*-positive breast cancer patients to tamoxifen is significantly worse than for HER-2/*neu*-negative patients [6,7] even though this point of view is not unopposed [8,9]. More recently, it was shown that aromatase inhibitors might provide more benefit than tamoxifen in patients with tumours positive for erbB-1 and/or erbB-2 [10].

However, the strongest evidence for a predictive role for HER-2/*neu *comes from several retrospective trials that consistently showed that HER-2/*neu*-positive patients responded better to an anthracycline-based therapy than to treatment with cyclophosphamide, methotrexate and fluorouracil [11-13]. Because of this predictive impact of HER-2/*neu*, many of the studies on the prognostic role of HER-2/*neu *cannot be reliably interpreted when the patients enrolled were treated in an adjuvant setting. To avoid this potential bias, we have performed a retrospective study on the prognostic impact of HER-2/*neu *in a historical cohort of node-negative T1/T2 breast cancer patients who were at that time not being treated in an adjuvant setting.

A point of utmost importance when assessing the utility of HER-2/*neu *as a prognostic factor is the technique of HER-2/*neu *testing. Principally, gene-based assays such as Southern blot analysis or fluorecence *in situ *hybridisation (FISH) have to be distinguished from assays that assess the level of protein expression, such as western blot analysis or immunohistochemistry (IHC). When analysing the published studies of HER-2/*neu *as a prognostic factor with regard to the technique used, Ross and colleagues [14,15] found that those studies that applied FISH to the assessment of gene amplification found an association of HER-2/*neu *with the prognosis of the patients, whereas studies that used IHC for the assessment of protein expression gave rather ambiguous results. Reasons for the apparently worse performance of IHC than that of FISH could be differences in the sensitivity of the applied antibodies [16] or the lack of a uniform scoring system in most of the older studies that used IHC. Standardisation of HER-2/*neu *testing has received considerably more attention in recent years owing to the advent of trastuzumab, a monoclonal antibody that affords prolonged survival in patients with metastatic breast cancer [17]. To improve the quality of IHC when testing for HER-2/*neu *status before starting a therapy with trastuzumab, a standardised scoring protocol was developed. On the basis of these findings, an algorithm incorporating FISH only in doubtful cases (2+ in IHC) was introduced.

The aim of our present study on 101 node-negative breast cancer cases was to compare gene amplification by FISH with protein expression by IHC with the standardised scoring system, the above-mentioned algorithm and the combination of HER-2/*neu *overexpression and amplification in the prognostic value of HER-2/*neu*.

## Methods

### Patients

The study cohort consisted of 101 lymph-node-negative breast cancer patients who were treated at the Department of Obstetrics and Gynecology of the Johannes Gutenberg University Mainz between 1988 and 1993. Patients were all treated with surgery and did not receive any systemic therapy in the adjuvant setting. The established prognostic factors (tumour size, histological grade and steroid receptor status) were collected from the original pathology reports of the gynaecological pathology division within our department.

Patients were treated either with modified radical mastectomy (*n *= 58) or breast-conserving surgery followed by irradiation (*n *= 43). Because the administration of adjuvant systemic therapy was not allowed in this study, we focused on node-negative breast cancer patients with pT1 and pT2 tumours and without any evidence of metastasis at the time of surgery. The median age of the patients at surgery was 56 years (range 29–86 years).

The median time of follow-up was 131 months for the patients still alive at the time of analysis. Within this follow-up period, 31 patients relapsed, 20 patients died of breast cancer and 10 patients died of unrelated causes. The patients dying of causes other than breast cancer were censored for the survival analyses at their date of death.

### HER-2/*neu *amplification determined by FISH

FISH for HER-2/*neu *gene amplification was performed with the Appligene Oncor HER-2/*neu *gene amplification system (Ventana Medical Systems, Tucson, AZ, USA), following the supplier's instructions. In brief, fresh frozen slides were first treated with a protein-digesting enzyme at 37°C for 10 min, washed in 2× sodium chloride/sodium citrate (SSC) at 22°C, dehydrated in an 75–100% ethanol series and air dried. Tissue sections were than denatured for 5 min in 70% formamide, pH 7.5, at 75°C, followed by rinsing with 100% ethanol and air drying. Appligene Oncor HER-2/*neu *DNA probe was prewarmed for 5 min at 37°C before application. Slides were than incubated for 24 hours at 37°C in a humidified chamber. After hybridisation, the slides were washed in 2× SSC for 5 min at 72°C; this was then followed by a wash in phosphate-buffered detergent (Oncor, Gaithersburg, MD, USA) at room temperature for 5 min. Detection was achieved with the Appligene Oncor fluorescein-labelled anti-digoxigenin antibody (Ventana Medical Systems). The slides were incubated with this antibody for 5 min in a humidified chamber at 37°C. Slides were then subjected to three washes (2 min each) in phosphate-buffered detergent at room temperature and were stored in the dark at -20°C for up to 5 days before analysis. The nuclei were counterstained with a propidium iodide/antifade solution (Oncor, Gaithersburg, MD, USA). Appropriate positive controls were included in each staining run. A serial section of each slide used for FISH was stained with haematoxylin and eosin to control for the presence of invasive tumour formations.

Additionally, serial sections (6 μm thick) of formalin-fixed paraffin-embedded blocks from five randomly selected amplified and five randomly selected non-amplified tumours were deparaffinised and then subjected to the staining protocol outlined above.

### Interpretation of FISH results

Analysis was performed with an Axioskop fluorescence microscope (Zeiss, Jena, Germany). Images were captured with an analogue camera (Leica, Bensheim, Germany). In each quadrant of the slide, the number of fluorescein signals was counted in 20 nuclei of invasive tumour cells (that is, a total of 80 tumour nuclei). Cases were considered amplified if the mean number of fluorescence signals was greater than four (HER2_AMP) [18]. Additionally, we compared tumours with low-level amplification (five or six signals per nucleus) against tumours with a higher level of amplification (more than six signals per nucleus).

### HER-2/*neu *expression determined by IHC

The immunohistochemical staining with a monoclonal antibody against HER-2/*neu *was performed as described previously [19]. In brief, serial sections (4 μm thick) of formalin-fixed, paraffin-embedded blocks were first deparaffinised. They were then microwaved in 10 mM citrate buffer, pH 6.0, to unmask epitopes and treated for 10 min with 1% hydrogen peroxide to block endogenous peroxidase. The sections were incubated for 30 min at 37°C with monoclonal HER-2/*neu *antibodies (clone CB-11; Novocastra, Newcastle upon Tyne, UK) diluted 1:50. The sections were then incubated with a biotin-labelled secondary antibody and streptavidin–peroxidase for 20 min each. Tissue was subsequently treated for 5 min with 0.05% 3',3-diaminobenzidine tetrahydrochloride and lightly counterstained with haematoxylin. All series included appropriate positive and negative controls. All controls gave adequate results.

### Interpretation of IHC results

Only cases showing unequivocal staining of membranes were regarded as positive for HER-2/*neu *overexpression [2]. A score was determined in accordance with the criteria used in the approval trials for trastuzumab [17,20]. In brief, cases showing no staining were scored 0, cases with less than 10% membrane staining 1+, cases with more than 10% weak to moderate complete membrane staining 2+, and cases with more than 10% strong complete membrane staining 3+ (HER2_SCO). Only 3+ cases were considered positive for survival analyses.

### Interpretation of combined IHC and FISH results

Cases that were scored 2+ by IHC were considered positive for the HER-2/*neu *algorithm [21] only if they showed an amplification of HER-2/neu (HER2_ALG).

Finally, cases showing HER-2/neu amplification as well as overexpression with an immunohistochemical score of 3+ were considered positive for the combined HER-2/*neu *evaluation (HER2_COM).

### Statistical analysis

The concordance between two methods was assessed by using the kappa test. The sensitivity and specificity of IHC (HER2_SCO) were evaluated, with the use of FISH as reference method. Life tables were calculated in accordance with the Kaplan–Meier method. Disease-free survival (DFS) was computed from the date of diagnosis to the date of recurrence of disease. Overall survival (OS) was computed from the date of diagnosis to the date of death from breast cancer. Patients who died of an unrelated cause were censored at the date of death. Survival curves were compared with the log-rank test. Univariate Cox survival analyses were performed and multivariate analyses were done in a backward stepwise fashion with the Cox proportional hazards model. All tests were performed at a significance level of α = 0.05. All *P *values are two-sided.

## Results

### Distribution of traditional and HER-2/*neu*-related factors

In a group of 101 node-negative patients with primary breast cancer of sizes T1 and T2 without systemic treatment in the adjuvant setting, established pathological and clinical parameters (tumour size, histological grade, steroid hormone receptor status, age and menopausal status) and HER-2/neu-related parameters (HER2_AMP, HER2_SCO, HER2_ALG and HER2_COM) were assessed and are presented in Table 1; 36% of the patients were premenopausal and perimenopausal. Tumour size was T1 in 57% and T2 in the remaining 43%. A total of 64% of the patients had tumours with a positive steroid hormone receptor status; that is, they were oestrogen receptor and/or progesterone receptor positive. A favourable histological grade (G I) was present in 17%, G II in 58% and G III in 20%. Five percent had medullary carcinomas and were therefore not assigned a histological grade.

HER-2/*neu *overexpression classified as 3+ as assessed by IHC was found in 20% (HER2_SCO); 17% of tumours showed an amplification of HER-2/*neu *by FISH (HER2_AMP). Of the amplified cases, five tumours showed five or six signals per nucleus, indicating low-level amplification, and 12 tumours showed more than six signals per nucleus (evidence of a higher level of amplification). Two of the patients who were scored as 2+ by IHC also exhibited an amplification of HER-2/*neu*. This resulted in a total of 22% of the patients being positive for HER2_ALG. Finally, in 11% of the patients an amplification as well as a 3+ overexpression of HER-2/*neu *was found (HER2_COM).

The estimated DFS was 70% and the breast cancer-specific OS was 80% at 10 years for the whole group of patients.

### Concordance of amplification status in formalin-fixed paraffin-embedded tumour samples with fresh-frozen tumour samples

All five tumours amplified for HER-2/*neu *in frozen tumour samples were also amplified when formalin-fixed paraffin-embedded tumour samples were used. Similarly, complete concordance was found between frozen and formalin-fixed tissue samples when five tumours without amplification were compared pair by pair.

### Concordance of HER2_AMP and HER2_SCO

A concordance between amplification and HER2_SCO was detected in 86 cases (85%) when only 3+ cases were considered positive for HER2_SCO. Six cases with amplification did not score 3+, whereas nine 3+ cases failed to show an amplification. This resulted in a degree of concordance (kappa) of 0.50 (95% CI 0.29–0.72).

### Sensitivity and specificity of HER2_SCO

The sensitivity of HER2_SCO 3+ with FISH as reference method was 65% (11 of 17 amplified cases) and specificity was 89% (75 of 84). Considering also 2+ cases as positive resulted in an increase in sensitivity to 76% (13 of 17 amplified cases) with a decreased specificity of 70% (59 of 84).

### Breast cancer-specific DFS

**Figure 1:**
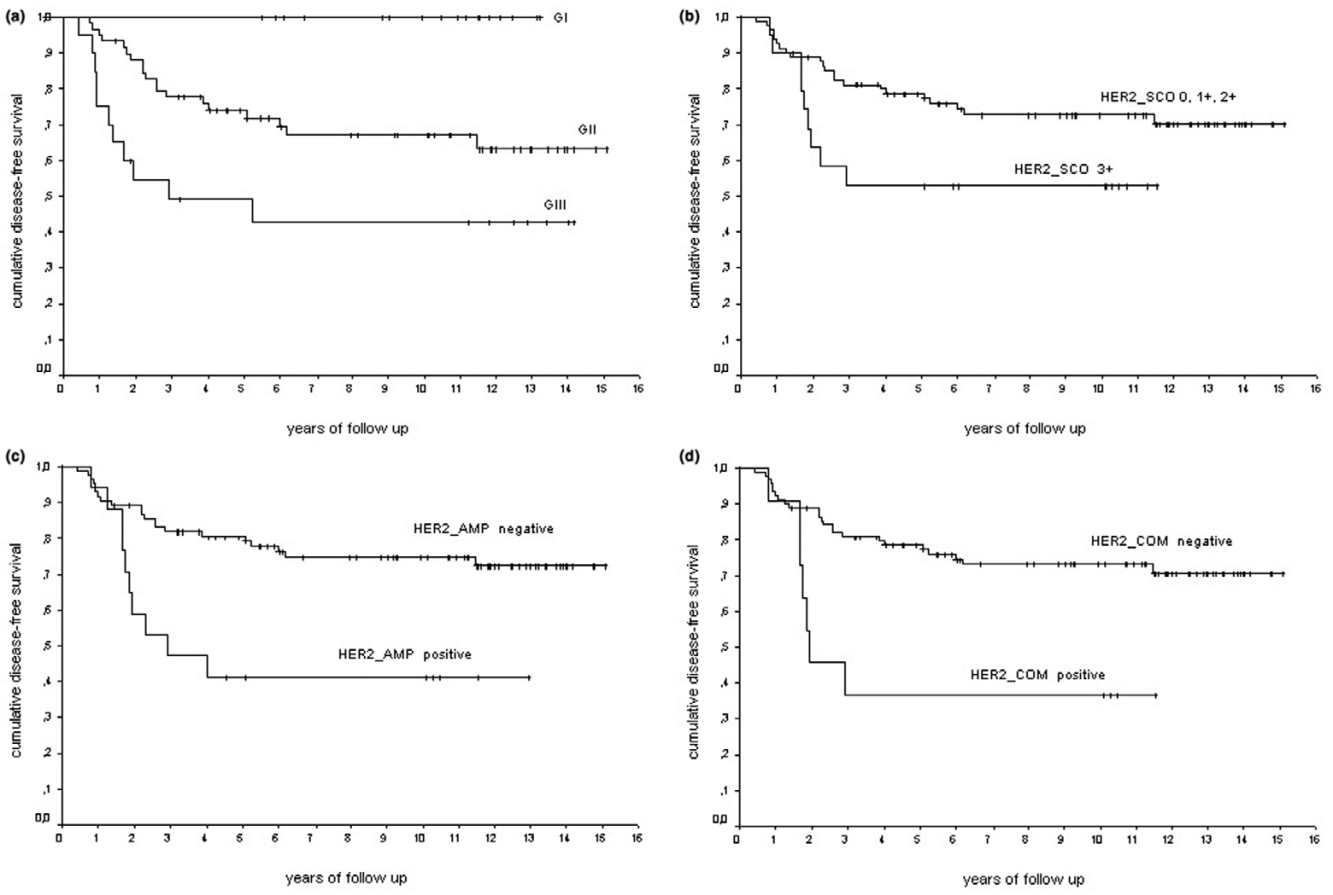
Effect of histological grade, HER2_SCO, HER2_AMP and HER2_COM on disease-free survival. **(a) **Grade; **(b) **HER2_SCO; **(c) **HER2_AMP; **(d) **HER2_COM.

In univariate analysis (Table 2) neither age at diagnosis nor menopausal status, tumour size or steroid hormone receptor status had a significant influence on the DFS. From the classical prognostic factors only histological grade turned out to be significantly related to DFS (*P *= 0.0002; RR 3.26, 95% CI 1.77–5.99) for DFS. Among the HER-2/*neu*-related variables, HER2_ALG did not show an influence on DFS, whereas HER2_SCO had a borderline significance (*P *= 0.059, RR 1.46, 95% CI 0.99–2.16). In contrast, both HER2_AMP (*P *= 0.004, RR 3.07, 95% CI 1.44–6.57) and HER2_COM (*P *= 0.006, RR 3.27, 95% CI 1.40–7.65) had a significant influence on the DFS. There was no significant difference in DFS between tumours with low-level amplification (five or six copies per nucleus) and tumours with a higher level of amplification. Three of five and 7 of 12 patients relapsed, respectively. The Kaplan–Meier estimates that yielded significant results are shown in Fig. 1. We then conducted a multivariate Cox regression in a backward fashion. In this Cox regression only histological grade (*P *= 0.0002, RR 3.22, 95% CI 1.73–5.99) and HER2_AMP (*P *= 0.026, RR 2.47, 95% CI 1.12–5.48) retained an independent prognostic significance (Table 3).

### Breast cancer-specific OS

**Figure 2:**
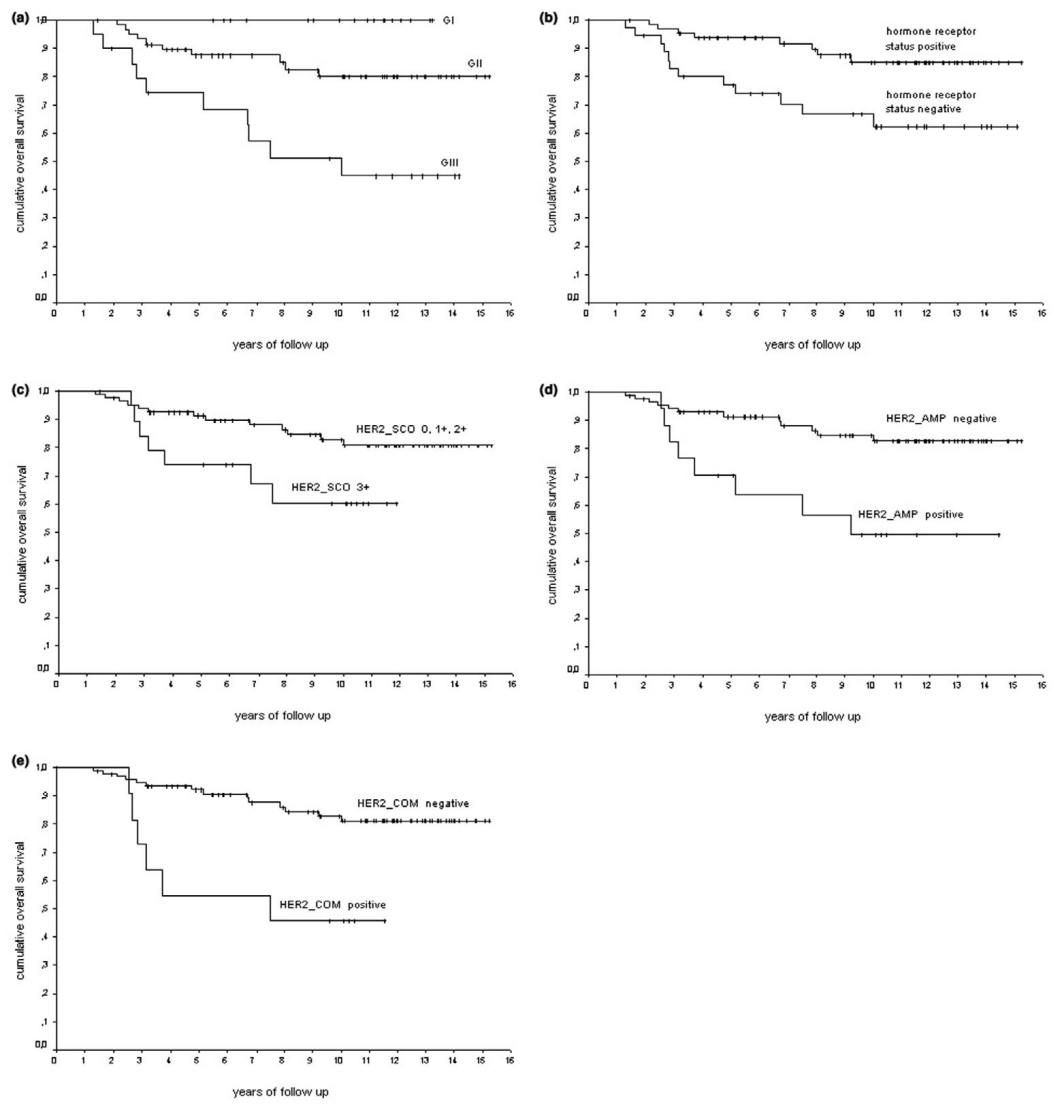
Effect of histological grade, steroid hormone receptor status, HER2_SCO, HER2_AMP and HER2_COM on overall survival. **(a) **Grade; **(b) **steroid hormone receptor status; **(c) **HER2_SCO; **(d) **HER2_AMP; **(e) **HER2_COM.

In univariate analysis (Table 4) neither age at diagnosis nor menopausal status or tumour size had a significant influence on the OS. From the classical prognostic factors only histological grade (*P *= 0.0003, RR 4.27, 95% CI 1.96–9.29) and the steroid hormone receptor status (*P *= 0.012, RR 0.32, 95% CI 0.13–9.78) were significant in univariate analysis for OS. Among the HER-2/*neu*-related variables, HER2_ALG did not show any influence on OS whatsoever. However, HER2_SCO (*P *= 0.047, RR 1.60, 95% CI 1.01–2.53) as well as HER2_AMP (*P *= 0.004, RR 3.78, 95% CI 1.54–9.26) and HER2_COM (*P *= 0.004, RR 4.18, 95% CI 1.60–10.90) had a significant influence on the OS. There was no significant difference in OS between tumours with low-level amplification (five or six copies per nucleus) and tumours with a higher level of amplification. Three of five and 5 of 12 patients died of breast cancer, respectively. The Kaplan–Meier estimates that yielded significant results are shown in Fig. 2. In a multivariate Cox regression analysis only histological grade (*P *= 0.0007, RR 3.89, 95% CI 1.77–8.55) and HER2_AMP (*P *= 0.016, RR 3.08, 95% CI 1.24–7.67) retained an independent prognostic significance (Table 5).

## Discussion

The prognostic value of HER-2/*neu *has always been controversial. Studies showing a shorter DFS and/or OS for HER-2/*neu*-positive patients [3,18,22,23] were opposed by studies which failed to find such an association [4,5]. In an earlier series from our department [24] an association with survival was shown only for node-positive, but not for node-negative, breast cancer patients. This association with prognosis in node-positive patients, who are almost uniformly treated with systemic therapy, might be markedly influenced by the ability of HER-2/*neu *status to affect responsiveness to systemic treatment [11-13]. In any case, the actual role of HER-2/*neu *as a predictive marker is still a matter of debate [25]. In our present study, we could rule out any such interference of purely prognostic with treatment-related predictive effects because we examined only node-negative patients without any systemic therapy in the adjuvant setting.

When examining the techniques used for the assessment of the HER-2/*neu *status, gene-based assays almost uniformly confirmed the negative prognostic impact of HER-2/*neu *in node-negative patients. Especially in the past few years, FISH has gained considerable interest as a reliable and valid method for determining the HER-2/*neu *status, confirming its prognostic utility [18,26]. Compared with other gene-based assays such as Southern blotting or polymerase chain reaction, FISH is not hampered by dilutional artefacts possibly resulting from a mixture of different cell populations. Similarly to IHC it allows for the specific detection of the alteration in individual cells within the important architectural context. However, in comparison with IHC, FISH is rather time-consuming and leads to substantial costs [27]. It is nevertheless considered the gold standard for assessing HER-2/neu status. A potential advance in the practicability of *in situ *hybridisation could be the more recently described chromogenic *in situ *hybridisation, which has shown a good correlation with FISH [28] and an independent prognostic importance in patients with node-negative breast cancer [23].

When determining the amplification of HER-2/*neu *with FISH in our cohort of 101 untreated node-negative breast cancer patients, we found 17% with an amplification of the gene locus. This fraction is largely in line with the literature [18,26,29-31]. The amplification of HER-2/neu measured by FISH showed a significant correlation with the DFS and OS. This strong association between amplification of HER-2/*neu *and survival of lymph-node-negative breast cancer patients is consistent with previous studies [18,23,26,30]. Only one group could document this association only for OS but not for DFS [32]. Searching for an explanation for their deviating results, the authors speculated that HER-2/*neu *might be more a predictive factor for treatment response than a prognostic factor *per se *because all patients in their study had been treated systemically after relapse.

In contrast with HER-2/*neu *gene amplification, overexpression of HER-2/*neu *was not associated with survival in several studies [4,5,33] even though others found a prognostic impact [22,23,34]. More recently, Volpi and colleagues [35] found a prognostic significance of HER-2/*neu *overexpression only for a subgroup of patients with high proliferative activity, whereas they failed to show any prognostic significance of HER-2/*neu *overexpression in the overall series of node-negative breast cancer patients. These apparent discrepancies have largely prevented the widespread acceptance of HER-2/*neu *as a prognostic factor in node-negative breast cancer up to now [36].

Several possible reasons could account for these controversial findings. One frequently quoted, simple but nonetheless unsatisfactory reason could be the rather small sample size of several studies. However, a strong and biologically relevant prognostic factor should eventually become evident even within a small sample size. Another possible reason is the remarkable discrepancy in sensitivity between the numerous antibodies used [16] and differences in tissue fixation and processing [37]. However, perhaps the most important reason is the lack of a standardised evaluation protocol in many of the older studies. The standardisation of IHC has become increasingly important since the successful use of trastuzumab (Herceptin™) in the treatment of HER-2/*neu*-overexpressing metastatic breast cancer [17,20,38]. The United States Food and Drug Administration has approved a standardised IHC kit (HercepTest™; Dako) with a detailed published scoring system ranging from 0 to 3+. However, the HercepTest has a rather low specificity, as outlined by Jacobs and colleagues [39]. The studies mentioned above that led to the approval of trastuzumab for HER-2/*neu*-positive metastatic breast cancer used a cocktail of different antibodies, one of which, the monoclonal antibody CB11, was used in our study.

When we adapted the scoring system defined for the HercepTest to the CB11 antibody we used in our study, we found a borderline significant correlation with survival.

To our opinion, the concordance between FISH and IHC was only moderate at best with a rate of 85%, even though we regarded only 3+ cases as overexpressing HER-2/*neu*. This level of concordance is in line with previously published results [40]. However, others found higher levels of concordance between FISH and IHC, ranging from 92% up to 98% [39,41,42].

When FISH results were compared with IHC data by using computer-assisted image analysis, we showed previously that concordance rates can be significantly improved when the IHC signal is corrected by the subtraction of non-specific cytoplasmic chromogen deposition [43], suggesting that only the strictly membrane-confined IHC signal can be considered truly positive when estimating HER-2/*neu *IHC slides. However, similarly to our findings others have failed to detect oncogene amplification by FISH in as many as 51% of tumours with strong 3+ staining [44]. Because overexpression of HER-2/*neu *is not necessarily caused only by amplification, polysomy of chromosome 17 has to be taken into account [45]. This polysomy of chromosome 17 could well lead to an incorrect diagnosis of a low-level amplification using single-colored FISH. However, in our study tumours with five or six copies of HER-2/*neu *had a survival comparable to that of tumours with a higher level of amplification. As a consequence of the moderate concordance, sensitivity and specificity for IHC in our study were lower than described by others [41,42]. However, our findings with the frequently used antibody CB11 should not be generalised to IHC as a whole because different antibodies show well-documented differences in terms of sensitivity and specificity [16].

Cases reported as 2+ by IHC should be reassessed with FISH in accordance with the test algorithm used in metastatic breast cancer before the start of a therapy with trastuzumab [21]. To the best of our knowledge we are the first group to use this algorithm to assess the prognosis in node-negative breast cancer patients. The use of this algorithm found 2 of 18 2+ tumours amplified and hence increased the percentage of tumours positive for HER-2/*neu *to 22%. A similar percentage of HER-2/*neu*-positive cases has previously been reported when performing FISH only in uncertain cases after IHC with the HercepTest [46]. For these authors, decreases in time and costs were quoted as strong arguments in favour of using this same testing algorithm for the determination of HER-2/*neu *status.

Because overexpression of HER-2/*neu *does not necessarily mirror amplification and vice versa, and because both parameters have been correlated with a poor outcome, we investigated whether the combination of amplification and overexpression could be useful in identifying a subgroup of patients with a particularly dismal prognosis. Altogether, 11 tumours showed amplification and overexpression (defined by a score of 3+). Indeed, these patients had a significantly worse prognosis than the remainder of the study cohort. These results are comparable to the data of Sauer and colleagues [31], who also found a markedly adverse prognostic effect in this dual-positive subgroup. Nonetheless, in our multivariate analysis, the combination of both techniques did not add additional prognostic information to that obtained by the amplification alone. However, owing to the relatively small number of events, especially for HER2_COM, subtle differences might be difficult to address adequately.

To assess the clinical relevance of these findings, we included the HER-2/*neu*-dependent variables (HER2_SCO, HER2_AMP, HER2_ALG and HER2_COM) into a multivariate model with the variables tumour size, histological grade and steroid receptor status. These last three variables are commonly accepted for risk assessment in node-negative breast cancer [36]. The factor age (not more than 35 years at diagnosis) was ignored because only one patient in our cohort was younger than 35. In this multivariate analysis, only histological grade and HER-2/*neu *amplification were identified as independent prognostic factors for DFS and OS, respectively.

We therefore conclude that HER-2/*neu *is an independent prognostic factor in node-negative breast cancer, even though its prognostic utility is largely influenced by the method of testing.

To further validate these observations, a prospective study in patients not treated in an adjuvant setting would be desirable. However, this is largely precluded in practice, given the recent consensus recommendations for the treatment of primary breast cancer [36]. For this reason we are currently engaged in a formal meta-analysis of all published studies that have used FISH or chromogenic *in situ *hybridisation to determine the HER-2/*neu *status and hope to clarify once and for all the controversial status of HER-2/*neu *as a prognostic factor in breast cancer.

## Conclusions

The prognostic significance of HER-2/*neu *in node-negative breast cancer depends strongly on the method of testing: only the amplification of HER-2/*neu *is an independent prognostic factor for the long-term prognosis of untreated node-negative breast cancer.

## Abbreviations

CI = confidence interval; DFS = disease-free survival; FISH = fluorescence *in situ *hybridisation; IHC = immunohistochemistry; OS = overall survival; RR = relative risk.

## Competing interests

The author(s) declare that they have no competing interests.

## Authors'contributions

MS planned the study, performed the IHC and drafted the manuscript. BL performed the *in situ *hybridisation. CG participated in the statistical analysis. NK participated in the *in situ *hybridisation. ES, HP, BT and WW participated in defining the study cohort and participated in the IHC. HAL and HK participated in finally drafting the manuscript. All authors read and approved the final manuscript.

## Figures and Tables

**Table 1:** Clinicopathological characteristics of 101 patients with node-negative primary breast cancer

Characteristic	*n*	%
Age at diagnosis		
Less than 50	36	36
More than 50	65	64
Menopausal status		
Pre/perimenopausal	36	36
Postmenopausal	65	64
pT stage		
pT1a,b	16	16
pT1c	42	42
pT2	43	43
Histological grade		
GI	17	17
GII	59	58
GIII	20	20
Not done (medullary carcinomas)	5	5
Steroid hormone receptor status		
Positive	65	64
Negative	36	36
HER2_SCO		
0, 1+	63	62
2+	18	18
3+	20	20
HER2_AMP		
Positive	17	17
Negative	84	83
HER2_ALG		
Positive	22	22
Negative	79	78
HER2_COM		
Positive	11	11
Negative	90	89

**Table 2:** Univariate analysis for breast cancer-specific disease-free survival

Prognostic factor	*P*	RR	95% CI
Age	n.s.	-	-
Menopausal status	n.s.	-	-
pT stage	n.s.	-	-
Grade	0.0002	3.35	1.77–5.99
Steroid hormone receptor status	n.s.	-	-
HER2_SCO	n.s.		
HER2_AMP	0.004	3.07	1.44–6.57
HER2_ALG	n.s.	-	-
HER2_COM	0.006	3.27	1.40–7.65

CI, confidence interval; n.s., not significant; RR, relative risk.

**Table 3:** Multivariate analysis for breast cancer-specific disease-free survival

Prognostic factor	*P*	RR	95% CI
Age	n.s.	-	-
Menopausal status	n.s.	-	-
pT stage	n.s.	-	-
Grade	0.0002	3.22	1.73–5.99
Steroid hormone receptor status	n.s.	-	-
HER2_SCO	n.s.	-	-
HER2_AMP	0.026	2.47	1.12–5.48
HER2_ALG	n.s.	-	-
HER2_COM	n.s.	-	-

CI, confidence interval; n.s., not significant; RR, relative risk.

**Table 4:** Univariate analysis for breast cancer-specific overall survival

Prognostic factor	*P*	RR	95% CI
Age	n.s.	-	-
Menopausal status	n.s.	-	-
pT stage	n.s.	-	-
Grade	0.0003	4.26	1.96–9.29
Steroid hormone receptor status	0.012	0.32	0.13–0.78
HER2_SCO	0.047	1.60	1.01–2.53
HER2_AMP	0.004	3.78	1.54–9.26
HER2_ALG	n.s.	-	-
HER2_COM	0.004	4.18	1.60–10.90

CI, confidence interval; n.s., not significant; RR, relative risk.

**Table 5:** Multivariate analysis for breast cancer-specific overall survival

Prognostic factor	*P*	RR	95% CI
Age	n.s.	-	-
Menopausal status	n.s.	-	-
pT stage	n.s.	-	-
Grade	0.0007	3.89	1.77–8.55
Steroid hormone receptor status	n.s.	-	-
HER2_SCO	n.s.	-	-
HER2_AMP	0.016	3.08	1.24–7.66
HER2_ALG	n.s.	-	-
HER2_COM	n.s.	-	-

CI, confidence interval; n.s., not significant; RR, relative risk.
